# Genomic and resistome characterization of a multidrug-resistant *Vibrio parahaemolyticus* outbreak clone linked to contaminated rice noodles

**DOI:** 10.3389/fmicb.2026.1871548

**Published:** 2026-06-25

**Authors:** Yuan Sun, Weihong Cui, Qiao Meng, Yangyang Liu, Wenjuan Liu, Yingchun Xu

**Affiliations:** 1 Shandong College of Traditional Chinese Medicine, Yantai, China; 2 Gaoxin Center for Disease Control and Prevention, Yantai, China; 3 Yantai Center for Disease Control and Prevention, Yantai, China

**Keywords:** core-genome multilocus sequence typing, cross-contamination, multidrug resistance, outbreak investigation, *Vibrio parahaemolyticus*, whole-genome sequencing

## Abstract

**Introduction:**

*Vibrio parahaemolyticus* is traditionally associated with raw seafood, but cross-contamination in commercial catering can create overlooked hazards. We investigated a gastroenteritis outbreak affecting 88 individuals to determine how a non-seafood vehicle became the route of transmission.

**Methods:**

We conducted integrated epidemiological and environmental traceback investigations. Whole-genome sequencing (WGS) and core-genome multilocus sequence typing (cgMLST) were applied to analyze the genetic relatedness and resistome of the recovered isolates.

**Results:**

Traceback strongly implicated rice noodles as the most likely vehicle. The outbreak was traced to a prerequisite program failure (raw clam exudate dripping onto uncovered noodles) followed by a critical control point failure (insufficient blanching for 1–2 min). WGS and cgMLST supported these findings: clinical and rice-noodle isolates were genetically identical (0–1 SNPs, *tdh*+), whereas a *V. parahaemolyticus* isolate from cooked bean sprouts was phylogenetically distant (>1300 allelic differences) and *tdh*-negative, excluding it as the cause. The outbreak clone also exhibited a multidrug-resistant (MDR) phenotype, including Colistin resistance and the *CARB-22 β-lactamase* gene.

**Conclusion:**

These results indicate that cross-contaminated food matrices can act as reservoirs for clinically relevant resistance determinants. This underscores the need for strict physical barriers, validated thermal processing, and high-resolution genomic surveillance to prevent opportunistic pathogen transmission in commercial catering.

## Introduction

*Vibrio parahaemolyticus* is a halophilic, Gram-negative bacterium globally recognized as a leading bacterial etiology of acute seafood-borne gastroenteritis (Baker-Austin et al., 2018; Wang et al., 2026). Epidemiological surveillance and genomic characterization have predominantly focused on the direct consumption of raw or undercooked marine products, which serve as the primary natural reservoirs for this pathogen. The clinical manifestation of the infection is primarily driven by well-characterized virulence factors, most notably the thermostable direct hemolysin (*tdh*) and the tdh-related hemolysin (*trh*) (Letchumanan et al., 2014; Raghunath, 2015). However, as global food supply chains expand and commercial catering scales up, the transmission paradigms of *V. parahaemolyticus* are shifting. Secondary transmission vehicles have emerged as a critical and often underestimated public health issue. For example, chemically neutral, non-seafood matrices, such as fresh produce or starch-based foods, may become inadvertently contaminated during food preparation (Berger et al., 2010; Malcolm et al., 2018). Such cross-contamination environments not only help pathogens survive outside marine ecosystems but also create complex transmission networks (Zhou et al., 2022).

Beyond its pathogenicity, antimicrobial resistance (AMR) in *V. parahaemolyticus* is a growing concern. Recent genomic surveillance shows an increasing number of multidrug-resistant (MDR) isolates, including resistance to extended-spectrum cephalosporins and last-resort drugs (Tan et al., 2020; Chen et al., 2024; Yan et al., 2024). In commercial kitchen environments, cross-contaminated food matrices can act as silent but potent reservoirs for these AMR determinants. The mobilization of resistome elements, particularly highly diverse β-lactamase genes such as blaCARB variants, through horizontal gene transfer (HGT) in these environments may increase the risk of dissemination (Elmahdi et al., 2016; Scapatico et al., 2021). Consequently, a localized failure in process hygiene can facilitate AMR transmission along the food chain, and may help explain why genotypic predictions sometimes do not match phenotypic resistance (Sebastian et al., 2025; Gomes et al., 2026).

In atypical foodborne outbreaks involving secondary vehicles, multiple isolates may be recovered from the implicated food matrix or its accompanying ingredients. In such settings, phenotypic typing or lower-resolution molecular methods, when used alone, may have limited discriminatory power for distinguishing outbreak-associated clones from incidental environmental isolates. WGS-based approaches, including wgSNP analysis and cgMLST, provide higher-resolution evidence for assessing genetic relatedness and have increasingly been used in foodborne outbreak investigations and source attribution. These methods are particularly useful when epidemiological traceback needs to be integrated with virulence profiles and antimicrobial-resistance determinants (Ronholm et al., 2016; Efsa Biohaz Panel et al., 2019; Yan et al., 2024; Zin et al., 2024). Unlike the conceptual review by Berger et al. (2010) or the simulation-based study by Malcolm et al. (2018), our study provides real-world outbreak evidence to address key questions that previous works could not: how WGS excludes environmental confounders in a true catering setting, how cascading PRP/CCP failures enable transmission through a non-seafood matrix, and what resistome architecture characterizes such an opportunistic clone. The precise genomic characterization of *V. parahaemolyticus* persisting and evolving in chemically neutral, high-starch matrices (such as rice noodles) is sparsely documented. cgMLST has rarely been applied in real-world cross-contamination scenarios to distinguish the true transmission vehicle from co-existing environmental strains that could otherwise mislead investigations. And the resistome architecture of these opportunistic outbreak clones, particularly the dissemination of diverse β-lactamase variants (e.g., *CARB* family) via non-seafood matrices, remains poorly profiled.

We investigated an atypical *V. parahaemolyticus* gastroenteritis outbreak that occurred at an industrial park canteen in Yantai, China, in September 2025, affecting 88 individuals and linked to a rice noodle dish. By integrating retrospective cohort data with high-resolution WGS, we aimed to: (1) resolve the genomic origin of the outbreak clone transmitted via this non-seafood vehicle; (2) use cgMLST to distinguish the *tdh*+ pathogenic cluster from confounding environmental isolates (recovered from bean sprouts); and (3) characterize the MDR profile and resistome architecture of the outbreak clone, including its *CARB* determinants.

## Materials and methods

### Epidemiological investigation

A retrospective cohort study was conducted among all 1,194 individuals who dined at the canteen during the implicated lunch period on September 3, 2025. Active case finding was initiated immediately upon notification on September 4. A case was defined as any individual who dined at the canteen on September 3, 2025, and developed diarrhea (≥3 times/day) with at least one accompanying symptom (abdominal pain, nausea, vomiting, fever, or headache). Cases were identified through multiple mechanisms: review of medical records from local hospitals, active symptom screening via questionnaires distributed to all employees, and monitoring of absenteeism records. Standardized data collection tools were used to obtain demographic information and detailed meal choices for all members. During the implicated lunch period on September 3, the canteen offered two mutually exclusive menu options, and employees self-selected one of the following: (1) the Rice Noodle Set (comprising rice noodles and cooked bean sprouts), or (2) the Standard Set (comprising tofu stew with clams, deep-fried pork tenderloin, stir-fried cabbage, steamed buns, and rice). This dichotomous meal selection provided a clear basis for defining the exposure groups in our retrospective cohort analysis. Attack rates and relative risks (RR) were calculated to compare the risk of illness between consumers of the Rice Noodle Set and the Standard Meal Set. A point-source epidemic curve was constructed to describe the temporal distribution of cases and estimate the incubation period.

### Food hygiene investigation

On September 4, the local CDC conducted a comprehensive on-site environmental assessment of the cafeteria. The investigation included a systematic inspection of all food processing and storage areas, observation of operational procedures, and in-depth semi-structured interviews with key kitchen personnel (including the head chef and cooks involved in preparing the suspected meal). The interviews focused on reconstructing the specific handling, storage, and cooking processes for the raw clams and rice noodles used on September 3. Documentation was reviewed to trace ingredient sources and comprehensively evaluate the entire preparation process of the rice noodle dish, with particular attention to spatial separation, equipment usage, and heat treatment procedures. Review of kitchen surveillance footage from September 3 confirmed these malpractices, specifically documenting the uncovered positioning of rice noodles directly beneath the perforated clam storage shelf and the brief (approximately 90 s) immersion of the noodles in boiling water.

### Sample collection and laboratory detection

A total of 54 samples were collected for microbiological analysis between September 4 and 5, 2025. These comprised 28 clinical specimens (stool, rectal swabs, vomitus) from symptomatic patients; 12 food samples (including rice noodles and their components, tofu stew with clams, and kitchen water, etc.); and 14 environmental swabs collected from food-contact surfaces, utensils, and food handlers’ gloves. Clinical specimens were placed in transport medium and maintained at 4 °C during transportation. All samples were processed within 4 h of collection.

All samples were examined for six major foodborne pathogens: *Staphylococcus aureus*, *Bacillus cereus*, *Salmonella* spp., diarrheagenic *Escherichia coli*, *Vibrio parahaemolyticus*, and *Burkholderia gladioli*. Isolation, identification, and confirmation procedures refereed to the standard protocols described in the U.S. Food and Drug Administration’s Bacteriological Analytical Manual (FDA BAM) (Food and Drug Administration [FDA], 2022) and, where applicable, relevant International Organization for Standardization (ISO) methods (International Organization for Standardization [ISO], 2004, 2012, 2017, 2021). For *V. parahaemolyticus*, species-specific confirmation was performed by PCR targeting the *toxR* gene (Kim et al., 1999). For *B. gladioli*, identification was further confirmed by species-specific PCR (Fiore et al., 2019; Furuya et al., 2020).

### Antimicrobial susceptibility testing

Antimicrobial susceptibility was determined using the broth microdilution method. A panel of 27 antibiotics was tested, including Ciprofloxacin (CIP), Ampicillin (AMP), Ampicillin-Sulbactam (AMS), Ceftazidime (CAZ), Ceftazidime/Clavulanic Acid (CAZ/C), Cefotaxime (CTX), Cefotaxime-Clavulanate (CTX/C), Cefixime (CFX), Cefpodoxime Proxetil (CPM), Nalidixic Acid (NAL), Chloramphenicol (CHL), Cefazolin (CFZ), Azithromycin (AZM), Ertapenem (ETP), Trimethoprim/Sulfamethoxazole (SXT), Ceftazidime/Avibactam (CZA), Tigecycline (TIG), Gentamicin (GEN), Tetracycline (TET), Meropenem (MEM), Amikacin (AMK), Amoxicillin-Clavulanate (AMC), Polymyxin B (PB), Imipenem (IPM), Streptomycin (STR), and Florfenicol (FFC). Minimum inhibitory concentrations (MICs) were interpreted according to Clinical and Laboratory Standards Institute (CLSI) guidelines M100 (34th Edition) (Clinical and Laboratory Standards Institute [CLSI], 2024) and M45-A3 (Clinical and Laboratory Standards Institute [CLSI], 2015). *Escherichia coli* ATCC 25922 served as the quality control strain.

### Whole-genome sequencing and bioinformatics analysis

Genomic DNA was extracted from six purified isolates (4 clinical, 1 rice noodle, 1 bean sprout) using the QIAamp DNA Mini Kit (Qiagen) and fragmented into 350 bp inserts using a Covaris ultrasonicator. Libraries were constructed using the TruSeq Library Construction Kit/MGIEasy FS DNA Prep kit and sequenced on the Illumina NovaSeq 6000 platform (PE150 mode) to achieve >1 Gb raw data per isolate. Raw sequence data were quality-controlled using FastQC v0.11.5 (Andrews, 2010) and Trimmomatic v0.36 (Bolger et al., 2014) integrated within the Microobench pipeline. *De novo* assembly was performed using SPAdes v3.13.0 (Bankevich et al., 2012), and the assembly quality was rigorously assessed with CheckM (Parks et al., 2015). Detailed assembly statistics, including total genome length, N50 value, contig count, completeness, and contamination percentages for all isolates, are provided in Supplementary Table 1.

Multilocus sequence typing (MLST) and core-genome MLST (cgMLST) were conducted utilizing PubMLST (Jolley et al., 2018) and the chewBBACA v2.8.5 pipeline (Silva et al., 2018). The cgMLST analysis was performed using the gcPathogen database schema encompassing 2635 loci. Virulence determinants were identified by aligning against the VFDB database (Version 2023-06) using DIAMOND v0.9.19 (Buchfink et al., 2015; Liu et al., 2019). Concurrently, antimicrobial resistance genes (ARGs) were predicted using the Comprehensive Antibiotic Resistance Database (CARD) and its Resistance Gene Identifier (RGI) v5.2.0 (Alcock et al., 2020).

Finally, high-resolution phylogenetic trees based on whole-genome SNPs (wgSNP) were constructed to determine the genetic relatedness of the isolates. SNPs were identified by mapping reads to the reference genome GCA_000196095.1 using the Microobench pipeline.

### Statistical analysis

All statistical data were managed and analyzed using R software version 4.3.1. Attack rates were calculated for each set meal served. Categorical variables were analyzed using Pearson’s chi-square test or Fisher’s exact test; the latter was specifically employed when expected cell counts were low (<5), such as in scenarios with zero cases in the unexposed group. For these zero-denominator scenarios, a Haldane-Anscombe continuity correction (adding 0.5 to all cells) was applied to calculate a finite Relative Risk (RR) with 95% confidence intervals (CIs). A two-sided *p*-value of <0.05 was considered statistically significant.

## Results

### Epidemiological findings

**FIGURE 1:**
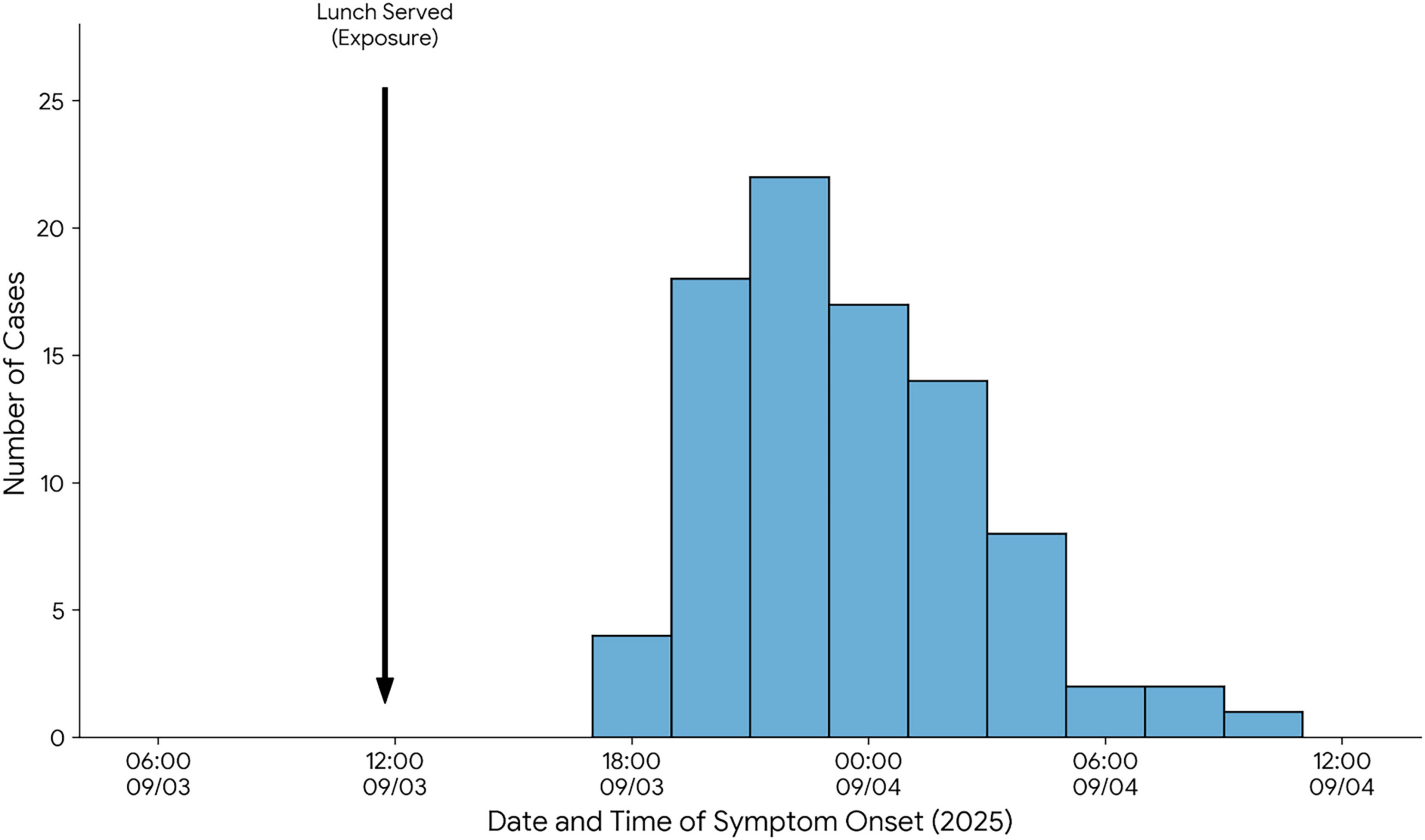
Epidemic curve of gastroenteritis cases (*n* = 88) by time of symptom onset. The outbreak shows a point-source exposure pattern. The implicated meal was served between 11:00 a.m. and 12:30 p.m. on September 3, 2025 (arrow). The median incubation period was 12.5 h.

Following the initial report of clustered gastroenteritis cases on September 4, 2025, a total of 88 individuals met the case definition, yielding an attack rate of 7.37% (88/1194) among the canteen patrons. The epidemic curve exhibited a unimodal distribution characteristic of a point-source exposure (Figure 1). The median incubation period was 12.5 h (range: 6.5–22.5 h). Patients exhibited typical gastroenteritis symptoms, predominantly diarrhea (100%), abdominal pain (75%), and vomiting (63.6%), consistent with an acute foodborne infection. Hematological analysis of 20 patients showed leukocytosis in 16 (80%), elevated neutrophil counts in 18 (90%), lymphocytopenia in 10 (50%), and increased C-reactive protein in 10 (50%). Fecal occult blood was positive in both patients tested.

A retrospective cohort analysis revealed a distinct separation in infection risk based on the patrons menu selections (Table 1). The attack rate for the Rice Noodle Set was 25.7% (88/343). Conversely, the Standard Set-which notably contained the suspected raw clam ingredient-had an attack rate of 0% (0/851). It established the non-seafood Rice Noodle Set as the most likely vehicle of infection (*p* < 0.001), indicating a massive cross-contamination event rather than primary contaminated ingredient consumption.

**TABLE 1:** Food-specific attack rates and risk estimation for the gastroenteritis outbreak.

Food item/ menu set	Ill (*n* = 88)	Well (*n* = 1106)	Total (*n* = 1194)	Attack rate (%)	Relative risk (95% CI)	*P*-value
Rice Noodle Set	88	255	343	25.7%	∞ (21.4–∞)^a^	<0.001
Standard Set	0	851	851	0.0%	Ref	–

Ref: Reference group.

^a^The Relative Risk (RR) utilizes a zero denominator because no cases occurred in the unexposed group. Calculated using the Haldane-Anscombe continuity correction: RR = ∞ (95% CI: 21.4–∞, *p* < 0.001).

### Environmental hygiene and traceback investigation

Field inspections and in-depth interviews with the kitchen staff revealed critical lapses in food handling protocols that facilitated cross-contamination. The investigation identified that raw clams were stored on a perforated shelf directly above an uncovered container of raw rice noodles. Kitchen staff admitted that exudate or thawing water from the raw seafood likely dripped onto the rice noodles below, establishing a direct transmission pathway. The interview disclosed that the rice noodles underwent an inadequate thermal processing step; they were blanched in boiling water for only 1–2 min to maintain texture. This brief exposure was insufficient to achieve the thermal lethality required to eliminate *V. parahaemolyticus*, thereby allowing the pathogen to persist in the served dish. Additionally, the investigation noted the cross-use of kitchen utensils, such as strainers and gloves, between the seafood and rice noodle preparation areas, further exacerbating the risk of contamination. Review of kitchen surveillance footage from September 3 confirmed these malpractices during food preparation.

### Microbiological identification and resistance

All clinical and food samples tested negative for *Staphylococcus aureus*, *Bacillus cereus*, *Salmonella* spp., diarrheagenic *Escherichia coli*, and *Burkholderia gladioli*, ruling out co-infections or other bacterial etiologies for this outbreak. Profiles *V. parahaemolyticus* was isolated from four patient samples (strains 2509S10019, 2509S10020, 2509S10021, 2509S10022), the raw rice noodles (strain 2509S10013), and the cooked bean sprouts (strain 2509S1003). Antimicrobial susceptibility testing identified two distinct phenotypes (Table 2): Cluster A (Patients & Rice Noodles): Isolates 2509S10013 and 2509S10019-22 exhibited a multidrug-resistant (MDR) profile, resistant to Cefazolin (MIC ≥ 8 μg/mL), Streptomycin (MIC ≥ 32 μg/mL), and Colistin (MIC ≥ 4 μg/mL), while remaining susceptible to all other tested antibiotics. Isolate B (Bean Sprouts): Strain 2509S1003 exhibited a broader resistance profile, including resistance to Ampicillin (MIC ≥ 32 μg/mL) and Polymyxin B (MIC ≥ 4 μg/mL), in addition to Cefazolin, Streptomycin, and Colistin, while remaining susceptible to all other tested antibiotics.

**TABLE 2:** Microbiological characteristics, antimicrobial resistance profiles, and virulence genotypes of *V. parahaemolyticus* isolates recovered from the outbreak.

Isolate ID	Source	Virulence genes	Phenotypic resistance profile (MIC in μ g/mL)^a^	Resistance genotypes	Cluster
2509S10019	Patient	*tdh*	CFZ (16), STR (4), CT (4)	*CARB-22*, *tet(35)*, *CRP*	Outbreak
2509S10020	Patient	*tdh*	CFZ (8), STR (4), CT (4)	*CARB-22*, *tet(35)*, *CRP*	Outbreak
2509S10021	Patient	*tdh*	CFZ (8), STR (4), CT (4)	*CARB-22*, *tet(35)*, *CRP*	Outbreak
2509S10022	Patient	*tdh*	CFZ (8), STR (4), CT (4)	*CARB-22*, *tet(35)*, *CRP*	Outbreak
2509S10013	Rice noodle	*tdh*	CFZ (8), STR (4), CT (4)	*CARB-22*, *tet(35)*, *CRP*	Outbreak
2509S1003	Bean sprouts	–	CFZ (16), STR (16), CT (8), AMP (32), PB (4)	*CARB-18*, *tet(35)*, *CRP*	Environmental

CFZ, Cefazolin; STR, Streptomycin; CT, Colistin; AMP, Ampicillin; PB, Polymyxin B.

^a^Resistance interpretation based on CLSI M45-A3 breakpoints.

### Genomic characterization

**FIGURE 2:**
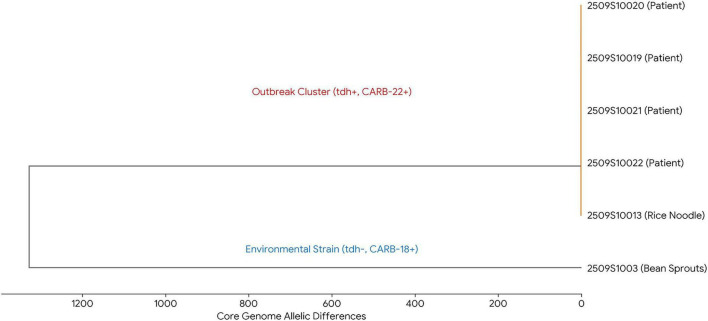
Core-genome phylogenetic tree showing the genetic relatedness among *Vibrio parahaemolyticus* isolates recovered from the outbreak. The tree was constructed based on core-genome allelic differences. The isolates from patients (2509S10019–2509S10022) and the implicated rice noodles (2509S10013) form a distinct, tightly clustered clonal lineage (Outbreak Cluster), exhibiting near-identical genetic profiles. In contrast, the isolate recovered from cooked bean sprouts (2509S1003) is located on a distant branch with >1300 allelic differences, identifying it as a genetically unrelated environmental strain. The scale bar represents the number of core genome allelic differences.

Whole-genome sequencing analysis provided strong genomic support for the epidemiological and environmental traceback. Phylogenetic Analysis: The wgSNP and cgMLST analysis demonstrated that the four patient isolates and the rice noodle isolate (2509S10013) were genetically identical, forming a tight clonal cluster (0-1 SNP differences). Conversely, the bean sprout isolate (2509S1003) was phylogenetically distant, differing by 1328 alleles in the core genome, indicating it was an unrelated environmental strain (Figure 2 and Table 2).

Virulence Factors: The outbreak cluster (Patients + Noodles) carried the *tdh* gene, a primary virulence marker for pathogenic *V. parahaemolyticus*. The bean sprout isolate (2509S1003) was *tdh*-negative, confirming its non-pathogenic status in this context.

**FIGURE 3:**
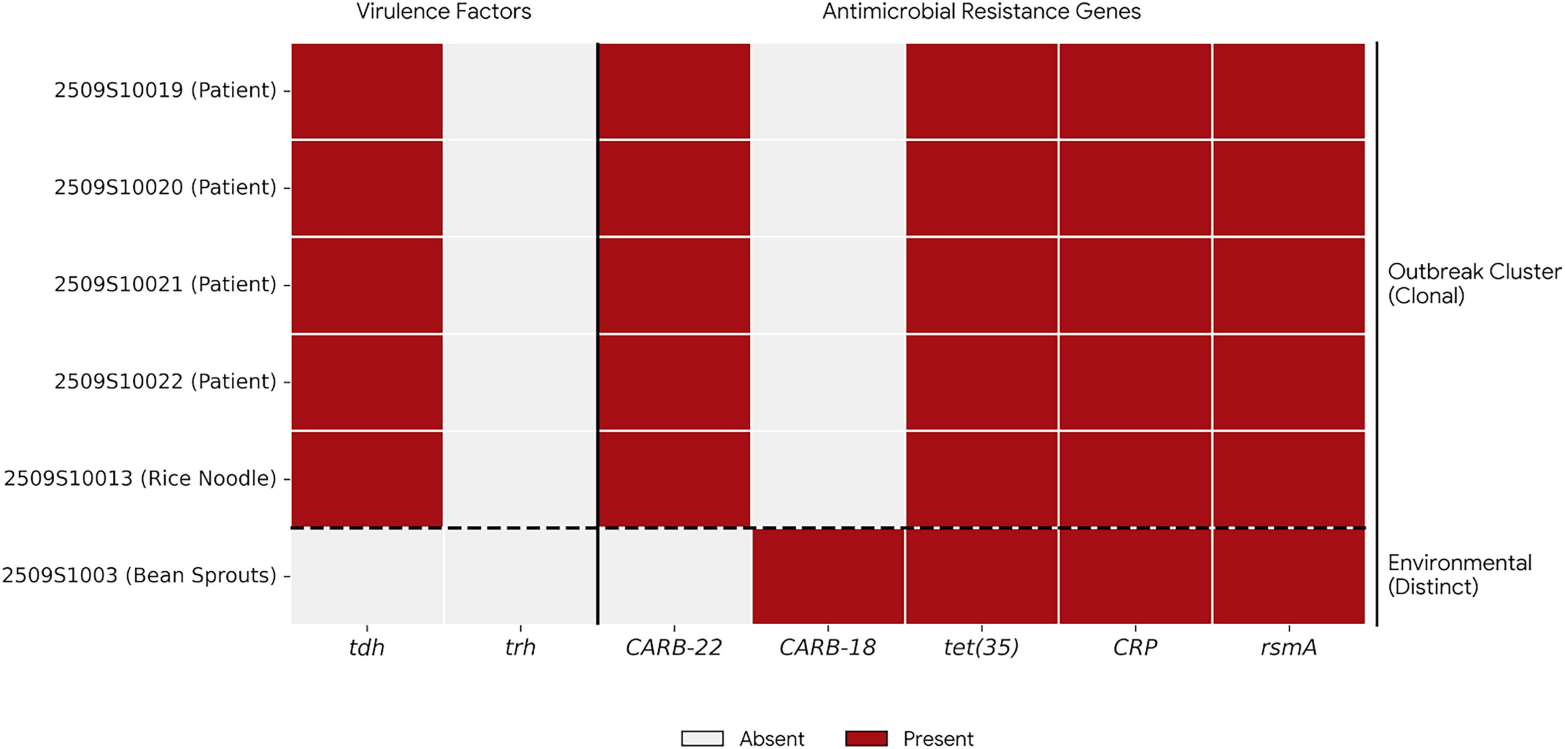
Heatmap profiling the distribution of key virulence factors and antimicrobial resistance genes across *Vibrio parahaemolyticus* isolates. Red squares indicate the presence of a gene, while gray squares indicate absence. The solid vertical line separates virulence determinants from resistance genes. The dashed horizontal line demarcates the genetically identical outbreak cluster (patient and rice noodle isolates) from the phylogenetically distinct environmental isolate recovered from bean sprouts. The outbreak cluster uniquely carries the pathogenicity marker *tdh* and the beta-lactamase gene *CARB-22*, whereas the environmental strain lacks tdh and carries a distinct beta-lactamase variant (*CARB-18*).

Resistance Genotypes: Genomic prediction of ARGs aligned with phenotypic data (Figure 3). All isolates carried CRP, rsmA, and tet(35). However, differences exist in the β-lactamase genes. The epidemic- and environment-associated clonal clusters carry the *CARB-18* and *CARB-22* variant genes, respectively, consistent with cefazolin resistance.

## Discussion

This study characterizes a *Vibrio parahaemolyticus* outbreak occurring in a corporate canteen in Yantai, China, affecting 88 individuals with an attack rate of 7.37%. Although the overall attack rate was relatively moderate compared with some outbreaks directly associated with raw seafood consumption, it remains epidemiologically important because the implicated vehicle was a secondary, non-seafood food matrix affected by cross-contamination. The epidemic curve displayed a classic point-source unimodal distribution with a median incubation period of 12.5 h. This timeframe aligns perfectly with the established pathophysiology of *tdh*-mediated gastroenteritis, wherein the thermostable direct hemolysin typically induces acute symptoms within 4–24 h post-ingestion (Baker-Austin et al., 2018).

Initial epidemiological investigations yielded a highly atypical transmission vehicle. In coastal regions of China, the vast majority of *V. parahaemolyticus* outbreaks are historically and definitively linked to the direct consumption of raw or undercooked marine products, such as oysters, sashimi, or crustaceans (Wu et al., 2014). In stark contrast, our food-specific attack rate analysis strongly exonerated the cooked clam dish served in the Standard Set and instead strongly implicated the Rice Noodle Set (*p* < 0.001). This critical finding challenges and expands traditional risk profiling paradigms, which frequently limit regulatory scrutiny to seafood-specific preparation stations. Our results underscore that in high-throughput commercial catering, starch-based, chemically neutral matrices like rice noodles can become potent secondary vehicles for marine halophilic pathogens when spatial separation from seafood handling is poorly managed.

Corroborating the epidemiological evidence, the subsequent environmental and operational traceback provided a clear mechanistic explanation for both the introduction and the persistence of the pathogen. Field inspections and personnel interviews revealed a critical failure in spatial segregation within the cold storage unit. Specifically, raw clams were stored on a perforated shelf directly above an uncovered container of raw rice noodles, establishing a definitive vertical cross-contamination pathway via dripping exudate. Given that filter-feeding bivalves can bioaccumulate *Vibrio* species at concentrations up to 100-fold higher than ambient seawater (Lopatek et al., 2018), this exudate likely introduced a massive bacterial load to the noodles below. This vertical transmission was further exacerbated by horizontal contamination; kitchen staff reported the cross-use of utensils, including strainers and gloves, between the seafood and rice noodle preparation zones. Following inoculation, pathogen survival was essentially guaranteed by a subsequent Critical Control Point (CCP) failure during thermal processing. The blanching process, which involved immersion in boiling water for only 1–2 min to maintain the texture of the noodles, was likely insufficient to inactivate *V. parahaemolyticus*. While *V. parahaemolyticus* is generally heat-sensitive, thermal inactivation kinetics typically indicate a *D*-value at 60 °C of approximately 2–5 min, depending on the food matrix (Lovingood et al., 2023). The brief exposure time, coupled with the transient temperature drop induced by submerging a large batch of cold noodles, failed to deliver the thermal energy required to achieve a standard 5-log reduction. Furthermore, the organic matter derived from the seafood exudate, combined with the high-starch matrix, likely offered additional protective effects for the bacteria to survive for extended periods (Burnett and Beuchat, 2001).

Ultimately, the resolution of this complex contamination scenario and the elimination of epidemiological confounding factors relied heavily on the application of Whole Genome Sequencing (WGS). Traditional culture methods had successfully isolated *V. parahaemolyticus* from both the implicated rice noodles and the cooked bean sprouts–a secondary component of the implicated dish. Relying solely on these conventional isolation techniques could have erroneously implicated the sprouts as the primary vehicle (Berger et al., 2010). However, high-resolution genomic analysis strongly refuted the sprout hypothesis. The core-genome phylogenetic tree demonstrated that the bean sprout isolate (2509S1003) was phylogenetically distant, exhibiting over 1300 core genome allelic differences compared to the clinical strains. Furthermore, it completely lacked the *tdh* virulence gene, characterizing it as a harmless, incidental environmental strain. Conversely, the isolate recovered from the rice noodles was genetically identical (0-1 SNPs) to the patient clinical isolates and carried the pathogenic *tdh* marker. This molecular identity strongly confirmed the cross-contaminated noodles as the outbreak vehicle, highlighting the unparalleled resolution of WGS over conventional phenotyping in modern outbreak investigations (Ronholm et al., 2016). Although cgMLST was sufficient in this investigation to separate the outbreak-associated clinical and rice-noodle isolates from the genetically distinct bean-sprout isolate, wgMLST may provide additional discriminatory power by incorporating a broader set of loci across the whole genome, including accessory genomic regions. In contrast, cgMLST focuses on conserved core-genome loci and therefore offers a more standardized and portable framework for comparing isolates across laboratories and datasets. In the present outbreak, the cgMLST result was concordant with the wgSNP analysis, which showed 0-1 SNP differences among the clinical and rice-noodle isolates and clear separation from the bean-sprout isolate. Therefore, wgMLST would be unlikely to alter the main epidemiological interpretation in this small outbreak dataset. Nevertheless, as sequencing capacity and allele-based genomic databases continue to improve, wgMLST may become increasingly useful for larger, prolonged, or multi-source outbreaks in which accessory-genome variation could provide additional resolution.

Beyond outbreak traceback, genomic profiling revealed a concerning multidrug-resistant (MDR) phenotype in the outbreak clone that warrants significant public health attention. The outbreak strain exhibited pronounced phenotypic resistance to Cefazolin, Streptomycin, and Colistin. The detection of the *CARB-22* beta-lactamase gene in the outbreak cluster, juxtaposed with *CARB-18* in the environmental strain, reflects the increasing and diverse dissemination of *CARB* family determinants in Asian aquaculture environments (Scapatico et al., 2021; Vandeputte et al., 2024). Of particular concern is the observed resistance to Colistin, a polymyxin antibiotic often considered a last-resort treatment. The presence of such resistance profiles in a foodborne pathogen suggests that *V. parahaemolyticus* can act as a silent reservoir for critical antimicrobial resistance determinants, which may transfer horizontally to the human gut microbiome after ingestion (Elmahdi et al., 2016). The presence of the *tdh* gene and absence of the *trh* gene in all clinical and noodle isolates further corroborated the pathogenic potential of this clone (Raghunath, 2015).

This study is subject to certain limitations. The retrospective cohort design inherently relies on participant recall, which can introduce bias regarding precise food consumption quantities. However, the stark “all-or-nothing” exposure pattern associated with the set menus largely mitigates this issue. Additionally, the original batch of raw clams was unavailable for direct microbiological sampling. Nevertheless, the convergence of observational kitchen data, robust epidemiological associations, and absolute genomic identity serves as definitive evidence. Furthermore, whole-genome sequencing was performed only on a single strain of *Vibrio parahaemolyticus* isolated from the contaminated rice noodles. Although the high degree of genomic similarity provides strong evidence, sequencing multiple strains from the food matrix would further enhance statistical rigor and improve the accuracy of phylogenetic analysis.

## Conclusion

This genomic study elucidates the latent transmission of *Vibrio parahaemolyticus* via chemically inert, non-seafood carriers, challenging traditional surveillance models centered on seafood. By applying cgMLST, we were able to distinguish the outbreak-associated, tdh-positive clonal cluster from the genetically distinct, tdh-negative environmental isolate recovered from bean sprouts, a distinction that standard phenotypic methods may not reliably achieve. Importantly, by identifying outbreak clones carrying multidrug resistance along with polymyxin resistance and the *blaCARB-22* determinant, we found that cross-contaminated food matrices serve not only as transient carriers but also as latent reservoirs for clinically significant resistant genomes. Together, these findings show that integrating whole-genome surveillance into routine outbreak investigations is essential not only for precise traceback but also for tracking and curbing the opportunistic spread of antimicrobial resistance in commercial catering.

## Data Availability

The relevant accession information is as follows: BioProject accession: PRJNA1452559 Linked BioSample accession numbers: SAMN57238043, SAMN57238044, SAMN57238045, SAMN57238046, SAMN57238047, SAMN57238048.

## References

[B1] Alcock B. P. Raphenya A. R. Lau T. T. Tsang K. K. Bouchard M. Edalatmand A. et al. (2020). CARD 2020: Antibiotic resistome surveillance with the comprehensive antibiotic resistance database. *Nucleic Acids Res.* 48 D517–D525. 10.1093/nar/gkz935 31665441 PMC7145624

[B2] Andrews S. (2010). *FastQC: A Quality Control Tool for High Throughput Sequence Data.* Cambridge: Babraham Bioinformatics, Babraham Institute.

[B3] Baker-Austin C. Oliver J. D. Alam M. Ali A. Waldor M. K. Qadri F. et al. (2018). *Vibrio spp*. infections. *Nat. Rev. Dis. Primers* 4 1–19. 10.1038/s41572-018-0005-8 30002421

[B4] Bankevich A. Nurk S. Antipov D. Gurevich A. A. Dvorkin M. Kulikov A. S. et al. (2012). SPAdes: A new genome assembly algorithm and its applications to single-cell sequencing. *J. Comput. Biol.* 19 455–477. 10.1089/cmb.2012.0021 22506599 PMC3342519

[B5] Berger C. N. Sodha S. V. Shaw R. K. Griffin P. M. Pink D. Hand P. et al. (2010). Fresh fruit and vegetables as vehicles for the transmission of human pathogens. *Environ. Microbiol.* 12 2385–2397. 10.1111/j.1462-2920.2010.02297.x 20636374

[B6] Bolger A. M. Lohse M. Usadel B. (2014). Trimmomatic: A flexible trimmer for Illumina sequence data. *Bioinformatics* 30 2114–2120. 10.1093/bioinformatics/btu170 24695404 PMC4103590

[B7] Buchfink B. Xie C. Huson D. H. (2015). Fast and sensitive protein alignment using DIAMOND. *Nat. Methods* 12 59–60. 10.1038/nmeth.3176 25402007

[B8] Burnett S. L. Beuchat L. R. (2001). Human pathogens associated with raw produce and unpasteurized juices, and difficulties in decontamination. *J. Ind. Microbiol. Biotechnol.* 27 104–110. 10.1038/sj.jim.7000199 11641768

[B9] Chen X. Zhang F. Lin G. Chen X. Huang H. Xu C. et al. (2024). Antibiotic resistance and genetic profiles of *Vibrio parahaemolyticus* isolated from farmed Pacific white shrimp (*Litopenaeus vannamei*) in Ningde regions. *Microorganisms* 12:152. 10.3390/microorganisms12010152 38257979 PMC10821069

[B10] Clinical and Laboratory Standards Institute [CLSI] (2015). *Methods for Antimicrobial Dilution and Disk Susceptibility Testing of Infrequently Isolated or Fastidious Bacteria*, 3rd Edn. Wayne, PA: CLSI. CLSI guideline M45.

[B11] Clinical and Laboratory Standards Institute [CLSI] (2024). *Performance Standards for Antimicrobial Susceptibility Testing*, 34th Edn. Wayne, PA: CLSI. CLSI supplement M100.

[B12] Efsa Biohaz Panel, Koutsoumanis K. Allende A. Alvarez-Ordóñez A. Bolton D. Bover-Cid S. et al. (2019). Whole genome sequencing and metagenomics for outbreak investigation, source attribution and risk assessment of food-borne microorganisms. *EFSA J.* 17:5898. 10.2903/j.efsa.2019.5898 32626197 PMC7008917

[B13] Elmahdi S. DaSilva L. V. Parveen S. (2016). Antibiotic resistance of *Vibrio parahaemolyticus* and *Vibrio vulnificus* in various countries: A review. *Front. Microbiol.* 7:571. 10.3389/fmicb.2016.00571 27052711

[B14] Fiore A. Lazzaro A. Gualandri V. (2019). A rapid method for the detection of *Burkholderia gladioli* in rice seeds. *J. Plant Pathol.* 101 801–805.

[B15] Food and Drug Administration [FDA] (2022). *Bacteriological Analytical Manual (BAM).* Silver Spring, MD: FDA.

[B16] Furuya N. Ura H. Iiyama K. Matsumoto M. Takeshita M. (2020). Development of specific PCR primers for identification of *Burkholderia gladioli*. *J. Gen. Plant Pathol.* 86 189–193.

[B17] Gomes E. Mesquita T. G. Serra P. Araújo D. Almeida C. Machado A. et al. (2026). Antimicrobial resistance in the food chain: Bridging knowledge gaps for effective detection and control. *Antibiotics* 15:262. 10.3390/antibiotics15030262 41892424 PMC13023466

[B18] International Organization for Standardization [ISO] (2004). *ISO 7932:2004. Microbiology of Food and animal Feeding Stuffs — Horizontal Method for the Enumeration of Presumptive Bacillus cereus.* Geneva: ISO.

[B19] International Organization for Standardization [ISO] (2012). *ISO/TS 13136:2012. Microbiology of Food and Animal Feed — Real-time Polymerase Chain Reaction (PCR)-based Method for the Detection of Food-Borne Pathogens.* Geneva: ISO.

[B20] International Organization for Standardization [ISO] (2017). *ISO 6579-1:2017. Microbiology of the Food Chain — Horizontal Method for the Detection, Enumeration and Serotyping of SALMONELLA — Part 1.* Geneva: ISO.

[B21] International Organization for Standardization [ISO] (2021). *ISO 6888-1:2021. Microbiology of the Food Chain — Horizontal Method for the Enumeration of Coagulase-Positive Staphylococci.* Geneva: ISO.

[B22] Jolley K. A. Bray J. E. Maiden M. C. (2018). Open-access bacterial population genomics: BIGSdb software, the website and their applications. *Wellcome Open Res.* 3:124. 10.12688/wellcomeopenres.14826.1 30345391 PMC6192448

[B23] Kim Y. B. Okuda J. Matsumoto C. Takahashi N. Hashimoto S. Nishibuchi M. (1999). Identification of *Vibrio parahaemolyticus* strains at the species level by PCR targeted to the toxR gene. *J. Clin. Microbiol.* 37 1173–1177. 10.1128/JCM.37.4.1173-1177.1999 10074546 PMC88669

[B24] Letchumanan V. Chan K. G. Lee L. H. (2014). *Vibrio parahaemolyticus*: A review on the pathogenesis, prevalence, and advance molecular identification techniques. *Front. Microbiol.* 5:705. 10.3389/fmicb.2014.00705 25566219 PMC4263241

[B25] Liu B. Zheng D. Jin Q. Chen L. Yang J. (2019). VFDB 2019: A comparative pathogenomic platform with an interactive web interface. *Nucleic Acids Res.* 47 D687–D692. 10.1093/nar/gky1080 30395255 PMC6324032

[B26] Lopatek M. Wieczorek K. Osek J. (2018). Antimicrobial resistance, virulence factors, and genetic profiles of *Vibrio parahaemolyticus* from seafood. *Appl. Environ. Microbiol.* 84:e00537-18. 10.1128/AEM.00537-18 29915109 PMC6070759

[B27] Lovingood A. M. Saldivar J. C. Lee J. (2023). Thermal inactivation of *Vibrio parahaemolyticus* in oyster meat. *J. Food Prot.* 86:100012. 10.1016/j.jfp.2022.10.005 36916595

[B28] Malcolm T. T. H. Chang W. S. Loo Y. Y. Cheah Y. K. Radzi C. W. J. W. M. Kantilal H. K. et al. (2018). Simulation of improper food hygiene practices: A quantitative assessment of *Vibrio parahaemolyticus* distribution. *Int. J. Food Microbiol.* 284 112–119. 10.1016/j.ijfoodmicro.2018.08.012 30142576

[B29] Parks D. H. Imelfort M. Skennerton C. T. Hugenholtz P. Tyson G. W. (2015). CheckM: Assessing the quality of microbial genomes recovered from isolates, single cells, and metagenomes. *Genome Res.* 25 1043–1055. 10.1101/gr.186072.114 25977477 PMC4484387

[B30] Raghunath P. (2015). Roles of Thermostable Direct Hemolysin (TDH) and TDH-related Hemolysin (TRH) in *Vibrio parahaemolyticus*. *Front. Microbiol.* 5:805. 10.3389/fmicb.2014.00805 25657643 PMC4302984

[B31] Ronholm J. Nasheri N. Petronella N. Pagotto F. (2016). Navigating microbiological food safety in the era of whole-genome sequencing. *Clin. Microbiol. Rev.* 29 837–857. 10.1128/CMR.00056-16 27559074 PMC5010751

[B32] Scapatico S. Smith C. J. Fanning S. (2021). The Vibrio spp. resistome: A comprehensive review. *Int. J. Antimicrob. Agents* 57:106345. 10.1016/j.ijantimicag.2021.106345 33887390

[B33] Sebastian P. J. Schlesener C. Byrne B. A. Miller M. Smith W. Batac F. et al. (2025). Antimicrobial resistance of *Vibrio spp*. from the coastal California system: Discordance between genotypic and phenotypic patterns. *Appl. Environ. Microbiol.* 91 e1808–e1824. 10.1128/aem.01808-24 39898660 PMC11921324

[B34] Silva M. Machado M. P. Silva D. N. Rossi M. Moran-Gilad J. Santos S. et al. (2018). chewBBACA: A complete suite for gene-by-gene schema creation and strain identification. *Microb. Genom.* 4:e000166. 10.1099/mgen.0.000166 29543149 PMC5885018

[B35] Tan C. W. Rukayadi Y. Hasan H. Thung T. Y. Lee E. Rollon W. D. et al. (2020). Prevalence and antibiotic resistance patterns of *Vibrio parahaemolyticus* isolated from different types of seafood in Selangor. *Malaysia. Saudi J. Biol. Sci.* 27 1602–1608. 10.1016/j.sjbs.2020.04.013 32489301 PMC7253911

[B36] Vandeputte M. Coppens S. Bossier P. Vereecke N. Vanrompay D. (2024). Genomic mining of *Vibrio parahaemolyticus* highlights prevalence of antimicrobial resistance genes and new genetic markers associated with AHPND and tdh+/trh+ genotypes. *BMC Genom.* 25:178. 10.1186/s12864-024-10093-9 38355437 PMC10868097

[B37] Wang J. Liu X. Yu L. Zhang M. Wei T. Li X. et al. (2026). Molecular epidemiological analysis of *Vibrio parahaemolyticus* in foodborne diseases in Liaoning province, 2023–2024. *Front. Public Health* 14:1780398. 10.3389/fpubh.2026.1780398 41988575 PMC13076565

[B38] Wu Y. Wen J. Ma Y. Ma X. Chen Y. (2014). Epidemiology of foodborne disease outbreaks caused by *Vibrio parahaemolyticus* in China, 2003–2008. *Food Control* 46 197–202. 10.1016/j.foodcont.2014.05.023

[B39] Yan W. Ji L. Dong F. Chen L. Yuan R. Zhang P. (2024). Antimicrobial resistance and genomic analysis of *Vibrio parahaemolyticus* isolates from foodborne outbreaks. Huzhou, China, 2019–2023. *Front. Microbiol.* 15:1439522. 10.3389/fmicb.2024.1439522 39323890 PMC11422088

[B40] Zhou H. Liu X. Hu W. Yang J. Jiang H. Sun X. et al. (2022). Prevalence, antimicrobial resistance and genetic characterization of *Vibrio parahaemolyticus* isolated from retail aquatic products in Nanjing. China. *Food Res. Int.* 162:112026. 10.1016/j.foodres.2022.112026 36461246

[B41] Zin H. Lim J. Shin Y. Kim B. Yoon M. Ha K. et al. (2024). Genomic insights into *Vibrio parahaemolyticus* from Southern Korea: Pathogenicity, antimicrobial resistance, and phylogenetic distinctions. *Microorganisms* 12:2497. 10.3390/microorganisms12122497 39770700 PMC11727765

