# Supplementary material for: Genomic and resistome characterization of a multidrug-resistant Vibrio parahaemolyticus outbreak clone linked to contaminated rice noodles
Source: Front Microbiol. 2026 Jun 25;17:1871548. doi: 10.3389/fmicb.2026.1871548 (PMC13346069; doi:10.3389/fmicb.2026.1871548)
Supplement: Supplementary file 1 [file Table_1.DOCX]

Table S1. Genomic assembly and quality assessment metrics of the six Vibrio parahaemolyticus isolates.

| **Strain ID** | **Source** | **Total assembly length (bp)** | **Number of scaffolds** | **N50 value (bp)** | **GC content (%)*** | **Completeness (%)** | **Contamination (%)** |
| --- | --- | --- | --- | --- | --- | --- | --- |
| **2509S10019** | **Patient (Clinical)** | **5120546** | **84** | **243223** | **45.32** | **100** | **0** |
| **2509S10020** | **Patient (Clinical)** | **5129172** | **93** | **243223** | **45.17** | **100** | **0** |
| **2509S10021** | **Patient (Clinical)** | **5134378** | **98** | **243755** | **44.98** | **100** | **0** |
| **2509S10022** | **Patient (Clinical)** | **5134378** | **98** | **243755** | **44.98** | **100** | **0** |
| **2509S10013** | **Rice noodles (Food)** | **5120546** | **84** | **243223** | **45.32** | **100** | **0** |
| **2509S1003** | **Bean sprouts (Environment)** | **5017769** | **52** | **491950** | **45.95** | **100** | **0.3** |
